# Quantitative phosphoproteomic analysis reveals reciprocal activation of receptor tyrosine kinases between cancer epithelial cells and stromal fibroblasts

**DOI:** 10.1186/s12014-018-9197-x

**Published:** 2018-06-15

**Authors:** Xinyan Wu, Muhammad Saddiq Zahari, Santosh Renuse, Nandini A. Sahasrabuddhe, Raghothama Chaerkady, Min-Sik Kim, Mary Jo Fackler, Martha Stampfer, Edward Gabrielson, Saraswati Sukumar, Akhilesh Pandey

**Affiliations:** 1 Department of Biological Chemistry, Johns Hopkins University, Baltimore, MD USA; 2 McKusick-Nathans Institute of Genetic Medicine, Johns Hopkins University, Baltimore, MD USA; 3 Institute of Bioinformatics, International Technology Park, Bangalore, 560066 India; 4 Manipal Academy of Higher Education, Manipal, Karnataka 576104 India; 5 Department of Oncology, Johns Hopkins University School of Medicine, Baltimore, MD 21205 USA; 6 Department of Pathology, Johns Hopkins University School of Medicine, Baltimore, MD 21205 USA; 7 Division of Biological Systems and Engineering, Lawrence Berkeley National Laboratory, Berkeley, CA USA; 8 Johns Hopkins University, 733 N. Broadway, Baltimore, MD 21205 USA

**Keywords:** Breast cancer, Epithelial cell, Carcinoma-associated fibroblast, Signaling crosstalk, SILAC, Phosphoproteome, Mass spectrometry, Co-culture

## Abstract

**Background:**

Cancer-associated fibroblasts (CAFs) are one of the most important components of tumor stroma and play a key role in modulating tumor growth. However, a mechanistic understanding of how CAFs communicate with tumor cells to promote their proliferation and invasion is far from complete. A major reason for this is that most current techniques and model systems do not capture the complexity of signal transduction that occurs between CAFs and tumor cells.

**Methods:**

In this study, we employed a stable isotope labeling with amino acids in cell culture (SILAC) strategy to label invasive breast cancer cells, MDA-MB-231, and breast cancer patient-derived CAF this has already been defined above cells. We used an antibody-based phosphotyrosine peptide enrichment method coupled to LC–MS/MS to catalog and quantify tyrosine phosphorylation-mediated signal transduction events induced by the bidirectional communication between patient-derived CAFs and tumor cells.

**Results:**

We discovered that distinct signaling events were activated in CAFs and in tumor epithelial cells during the crosstalk between these two cell types. We identified reciprocal activation of a number of receptor tyrosine kinases including EGFR, FGFR1 and EPHA2 induced by this bidirectional communication.

**Conclusions:**

Our study not only provides insights into the mechanisms of the interaction between CAFs and tumor cells, but the model system described here could be used as a prototype for analysis of intercellular communication in many different tumor microenvironments.

## Background

An intrinsic feature of cancers is the presence of a stromal compartment that generally provides a supportive microenvironment for tumor epithelial cells. It is becoming increasingly clear that malignant cells themselves are not sufficient to maintain tumor growth and progression and that the tumor stroma plays an essential role in this process [1, 2]. As a major component of the stromal compartment in tumors, fibroblasts can be activated and acquire a modified phenotype similar to fibroblasts associated with wound healing [1]. These activated cancer-associated fibroblasts (CAFs) are a major source of paracrine growth factors, including bFGF, HGF, and TGF-β, that can further promote the growth of carcinomas and recruit endothelial cells for angiogenic processes [1, 2].

It has been shown that CAFs isolated from various cancers, including breast, ovarian and prostate cancers, are more competent than normal fibroblasts in enhancing tumor growth [3]. CAFs have also been shown to promote genomic instability, induce epithelial-mesenchymal transition, and promote tumor growth and angiogenesis [4, 5]. Further, studies of bone marrow-derived tumor stromal cells have demonstrated that they are critical in creating pre-metastatic niches to facilitate the formation of metastases [6, 7]. These findings confirm that CAFs indeed have properties that are distinguishable from normal fibroblasts and are intimately involved in almost every step of tumor progression, from initiation to metastasis. The crucial role of fibroblasts in tumor development was recently demonstrated by a study showing that a signature derived from gene expression profiling of microdissected stroma from primary tumors, termed “stroma-derived prognostic predictor” (SDPP), could be used as a robust and accurate tool in predicting breast cancer prognosis [8]. Notably, this signature had an even higher accuracy than all other current mRNA-based profiles for prediction purposes, independent of clinical breast cancer subtypes such as ER and HER2 status [8]. One possible reason for such accuracy is that alterations in fibroblasts might be more uniform than those in carcinoma cells. Unlike the heterogeneous genomic and epigenetic alterations observed in breast cancer cells, which makes treatment of breast cancer challenging, the relative molecular uniformity of CAFs may lend itself to the development of optimal therapies for the prevention of metastasis.

Nevertheless, in order to target CAFs for cancer therapy, two major questions remain to be answered: What are the events that occur during conversion of normal fibroblasts to CAFs and what are the key mechanisms of interaction between CAFs and tumor cells that promote tumor aggressiveness. One approach to studying these interactions is by analyzing the conditioned media from cultured CAFs; indeed, growth factor-induced paracrine communication has been identified as one of the major mechanisms mediating the crosstalk between tumor cells and CAFs [4, 9, 10]. However, given that tumor cells physically and persistently interact with stromal cells in vivo, more complex communication events take place than those initiated by the soluble factors secreted into the extracellular environment. To this end, 2D and 3D co-culture systems have been developed to simulate the in vivo tumor microenvironment. However, conventional molecular biology approaches are not capable of discriminating whether the source of proteins is from the epithelial tumor cells or CAFs when they are co-cultured and processed together. This significantly limits their utility for systematic analysis of the true crosstalk between tumor epithelium and CAFs.

A mass spectrometry-based quantitative proteomic method, stable isotope labeling by amino acids in cell culture (SILAC), is an approach that utilizes amino acids with substituted stable isotopic nuclei to metabolically label whole cellular proteomes during cell culture [11–13]. Using this strategy, we can label the entire proteomes of different cells with stable isotope-containing amino acids, culture the cells together and still be able to distinguish the specific cellular source of proteins. SILAC labeling strategy has been used to identify bidirectional cell-specific signaling events initiated between HEK293 cells either expressing the Eph receptor, EPHB2 or its transmembrane ephrin ligand, ephrin-B1 [14]. More recently, this strategy was also employed to study the signaling crosstalk between MDA-MB-231 and human umbilical vein endothelial cells [15]. In additional to the classic SILAC based labeling strategy, another alternative cell-specific isotopic labeling technology, named cell-type-specific labeling with amino acid precursors has been developed [16] and used to study long-term signaling crosstalk between a human breast cancer cell line MDA-MB-231 and a mouse embryo fibroblast cell line, C3H/10T1/2 [17].

In this study, we employed the SILAC labeling strategy to differentially label a highly aggressive triple negative breast cancer cell line, MDA-MB-231 and a primary cultured human breast CAF (82T) during a short-term co-culture. Using antibody-based tyrosine phosphopeptide enrichment coupled with high-resolution and high-accuracy mass spectrometry, we systematically quantified the bidirectional phosphotyrosine proteome changes induced by the interaction of epithelial tumor cells and CAFs. We found differential activation of a number of tyrosine kinases when tumor cells were co-cultured with primary breast CAFs indicating that there are distinct signaling events occurring in epithelial tumor cells and in CAFs during such interactions that could play important roles in tumor development.

## Methods

### Cell culture and reagent

MDA-MB-231 cells were purchased from ATCC. Eight breast cancer associated fibroblast cell strains, 76T (p9), 72T (p7), 8T (p7), 85T (p5), 35T (p6), 84T (p6), 82T (p6) and 120T (p8) were generated by one of us (MS). MDA-MB-231 and all eight CAFs cell strains were cultured in DMEM-F12 medium supplemented with 5% FBS or 10% FBS, respectively. All cells were grown in 5% CO_2_ at 37 °C. DMEM-F12 with and without Lysine and Arginine, fetal bovine serum (FBS), l-glutamine, and antibiotics were purchased from Invitrogen (Carlsbad, CA). SILAC amino acids, ^2^H_4_-Lys and ^13^C_6_-Arg and heavy amino acids, ^13^C_6_^15^N_2_-Lys and ^13^C_6_^15^N_4_-Arg were purchased from Cambridge Isotope Laboratories (Andover, MA). (Andover, MA). Anti-phosphotyrosine mouse mAb (pTyr-100) beads were purchased from Cell Signaling Technology (Danvers, MA). TPCK-treated trypsin was obtained from Worthington Biochemical Corp. (Lakewood, NJ). All other reagents used in this study were from Fisher Scientific (Pittsburgh, PA).

### Retroviral and lentiviral production and cell infection

Retroviral expression plasmid, pKMRV-EGFP was co-transfected with the packaging vector, pCL-Ampho into HEK293T cells. Lentiviral plasmid expressing tdTomato Red, pFUtdTW (Addgene) was co-transfected with the helper plasmid pHR’8.2delta and the envelop plasmid, pCMV-VSV-G into HEK293T cells. Transfection is mediated by Lipofectamine 2000. pKMRV-EGFP and pFUtdTW viral supernatants were collected at 24, 48 and 72 h post transfection and used to infect 82T and MDA-MB-231 cells, respectively. Infected cells with strong expression of GFP or tdTomato Red were sorted using flow cytometry and expanded in cell culture for co-culture analysis.

### Cell proliferation analysis

A commonly used cell proliferation assay employing crystal violet dye [18, 19] was used to evaluate the cell growth rate for 8 CAFs. Briefly, 10,000 cells were seeded in each well of 24-well plates and cultured for 7 days. Cells were fixed and stained with 0.05% crystal violet in 4% formalin. Crystal violet dye in stained cells was then eluted with 0.4% acetic acid and measure at O.D. 595. To examine the cell growth in co-culture system, 82T CAFs or MDA-MB-231 breast cancer cells labeled with EGFP or tdTomato Red were seeded separately or in combination into each well of 96-well culture dishes and cultured in DMEM-F12 with 5% FBS. After 5 day, EGFP or tdTomato Red fluorescence was measured using a POLARstar Omega microplate plate reader.

### Soft agar colony formation analysis

Agar (0.5% bottom layer) was prepared in 6-well plates. Five hundred 82T or MDA-MB-231 cells were, separately or in combination, mixed with 0.35% agar in 1 × DMEM-F12 medium supplemented with 5% FBS and seeded on top of bottom layer agar. 1 ml of DMEM-F12 medium with 5% FBS was added in each well. Cells were grown for 14 days at 37 °C. Colonies were then stained with crystal violet and counted under the microscope. Colonies with size of more than 50 cells were counted.

### Immunoblot analysis

Individually cultured and co-cultured cells were harvested and lysed in modified RIPA buffer (50 mM Tris–HCl, pH 7.4, 150 mM NaCl, 1 mm EDTA, 1% Nonidet P-40, 0.25% sodium deoxycholate, and 1 mM sodium orthovanadate in the presence of protease inhibitors). 80 µg protein lysates were denatured and separated in NuPAGE gels (Invitrogen), transferred to nitrocellulose membranes and probed with HRP conjugated pY100 antibody (Cell Signaling Technology).

### Cell line SILAC labeling

Only non-transduced MDA-MB-231 and 82T cells were used for SILAC labeling and phosphoproteomics analysis. Three-state stable isotopic labeling by amino acids in cell culture (SILAC) of MDA-MB-231 and 82T was performed as described earlier [12, 20]. Briefly, to facilitate the incorporation of medium and heavy labels for MDA-MB-231 cells, MDA-MB-231 were cultured in DMEM-F12 SILAC labeling media supplemented with medium (^2^H_4_-Lys and ^13^C_6_-Arg) or heavy amino acids (^13^C_6_^15^N_2_-Lys and ^13^C_6_^15^N_4_-Arg). To facilitate the incorporation of light and heavy labels of 82T cells, the CAFs were cultured in DMEM-F12 media with light amino acids or heavy amino acids (^13^C_6_^15^N_2_-Lys and ^13^C_6_^15^N_4_-Arg). To achieve > 95% labeling efficiency, cells were cultured in corresponding SILAC media for at least 5 passages and labeling efficiency was assessed by LC–MS/MS analysis before any proteomics analysis.

### SILAC labeled CAFs and MDA-MB-231 co-culture

Nine million medium or heavy labeled MDA-MB-231 cells were seeded into each of 15 cm culture dish and cultured in corresponding SILAC media overnight. Next day, light or heavy labeled 82T cells were treated with 4 ml enzyme free Cell Dissociation Buffer (Invitrogen) to detach and dissociate cells. Cells were washed with cold PBS for three times and re-suspended in corresponding light or heavy DMEM-F12 SILAC media with 5% FBS. 9 million light labeled 82-L cells were seeded onto the plate with the pre-seeded medium labeled MDA-MB-231-M cells which were prewashed with cold PBS. The mixed cells were kept in heavy DMEM-F12 SILAC media with 5% FBS at 37 °C for 30 min. 9 million heavy labeled 82T-H cells were seeded onto new 15 cm culture dish in heavy SILAC media with 5% FBS at 37 °C for 30 min. In order to keep the individually cultured epithelial cells, MDA-MB-231 under the same treatment conditions, medium labeled MDA-MB-231 cells were also washed with cold PBS and incubated in SILAC DMEM-F12 medium media with 5% FBS at 37 °C for 30 min. These individually cultured heavy labeled MDA-MB-231-H and 82T-H cells served as reference controls for co-cultured MDA-MB-231-M cells and 82T-L cells. 82T cells attached during the 30 min of incubation. Both individually cultured and co-cultured cells were quickly washed with cold PBS once and harvested with 9 M urea lysis buffer (20 mM HEPES pH 8.0, 9 M urea, 1 mM sodium orthovanadate, 2.5 mM sodium pyrophosphate, 1 mM ß-glycerophosphate and 5 mM sodium fluoride).

### In-solution trypsin digestion

After cells were harvested using urea lysis buffer, lysates from co-cultured 82T-L and MDA-MB-231-M cells were mixed with lysates from individually cultured 82T-H cells or MDA-MB-231-H cells. After mixing, cell lysates were sonicated and then cleared by centrifugation at 3000×*g* at 4 °C for 10 min. As determined by BCA assay, 30 mg protein from mixed cell lysates was then reduced with 5 mM dithiothreitol and alkylated with 10 mM iodoacetamide. For in-solution tryptic digestion, 20 mM HEPES pH 8.0 was used to dilute the mixed cell lysates to the final concentration of urea lower than 2 M. The diluted cell lysates were then digested with 1 mg/mL TPCK-treated trypsin on an orbital shaker at 25 °C overnight. Tryptic peptides were acidified with 1% trifluoroacetic acid (TFA) and desalted using SepPak C_18_ cartridge. Eluted peptides were lyophilized to dryness prior to phosphotyrosine peptide enrichment.

### Immunoaffinity purification of phosphotyrosine peptides

Immunoaffinity purification (IAP) of phosphotyrosine peptides was performed as described [21]. Briefly, following lyophilization, desalted lyophilized tryptic peptides were reconstituted in 1.4 mL of IAP buffer (50 mM MOPS pH 7.2, 10 mM sodium phosphate, 50 mM NaCl). The reconstituted peptide mixtures were then incubated with anti-phosphotyrosine antibody beads (pY100, Cell Signaling Technology) on a rotator at 4 °C for 30 min. After incubation, phosphotyrosine peptides and the pY100 antibody complex were washed thrice with IAP buffer and then twice with water. Residual water was removed completely. Phosphopeptides were eluted from the antibody beads by acidifying the bead mixture at room temperature with 0.1% TFA. Phosphopeptides eluents were desalted with C_18_ STAGE tips, vacuum dried and stored at − 80 °C prior to LC–MS/MS analysis.

### Liquid chromatography tandem mass spectrometry

Data dependent LC–MS/MS analysis of phosphopeptides enriched by IAP was performed with an LTQ-Orbitrap Velos mass spectrometer (Thermo Fisher Scientific) coupled to a nano-liquid chromatography system (Proxeon, Easy Nano-LC). During each LC–MS/MS run, 1 μL of reconstituted peptide solution were injected onto a nano-C_18_ reversed phase column (10 cm × 75 µm, Magic C_18_ AQ 5 µm, 120 Å). Peptides were than fractionated across a 90-min linear reversed phase HPLC gradient (from 5 to 60% Acetonitrile). High-resolution precursor scans (FTMS) were acquired within the Orbitrap analyzer across a mass range of 350–1700 Da (with 60,000 resolution at 400 *m*/*z*). The ten most abundant precursor ions from each precursor scan were selected for high energy collision dissociation fragmentation (isolation width of 1.90 *m*/*z*; 35% normalized collision energy and activation time of 0.1 ms). High-resolution MS/MS spectra were acquired (at 15,000 resolution at 400 *m*/*z*) on the Orbitrap analyzer following fragmentation.

### Mass spectrometry data analysis

Proteome Discoverer (v2.0; Thermo Fisher Scientific) software package was used to facilitate downstream protein identification and quantitation. All acquired mass spectrometric data were searched within the Proteome Discoverer interface using the SEQUEST search algorithm against Human RefSeq database v 69 (containing 33,249 entries). The search parameters were as follows: a maximum of one missed cleavage; a fixed modification of carbamidomethylation; variable modifications of N-terminal acetylation, oxidation at methionine, phosphorylation at serine, threonine and tyrosine and SILAC labeling ^13^C_6_,^15^N_2_-lysine; ^2^H_4_-lysine; ^13^C_6_-arginine and ^13^C_6_,^15^N_2_-arginine; MS tolerance of ± 10 ppm; MS/MS tolerance of ± 0.1 Da. The SEQUEST score cut-offs were set to a false discovery rate of 1% at the peptide level. The probability that an identified phosphorylation was modifying each specific Ser/Thr/Tyr residue on each identified phosphopeptide was determined from the PhosphoRS algorithm [22]. We averaged the intensities of the phosphopeptides identified in the two biological replicate experiments that were carried out. A twofold cut-off was selected for hyperphosphorylation and a 0.5-fold cut-off was selected to denote hypophosphorylation. All mass spectrometry proteomics data associated with this project have been deposited to the ProteomeXchange Consortium (http://proteomecentral.proteomexchange.org) via the PRIDE partner repository with the dataset identifier PXD003544.

## Results

### Culture of primary tumor derived cancer associated fibroblasts

**Fig. 1:**
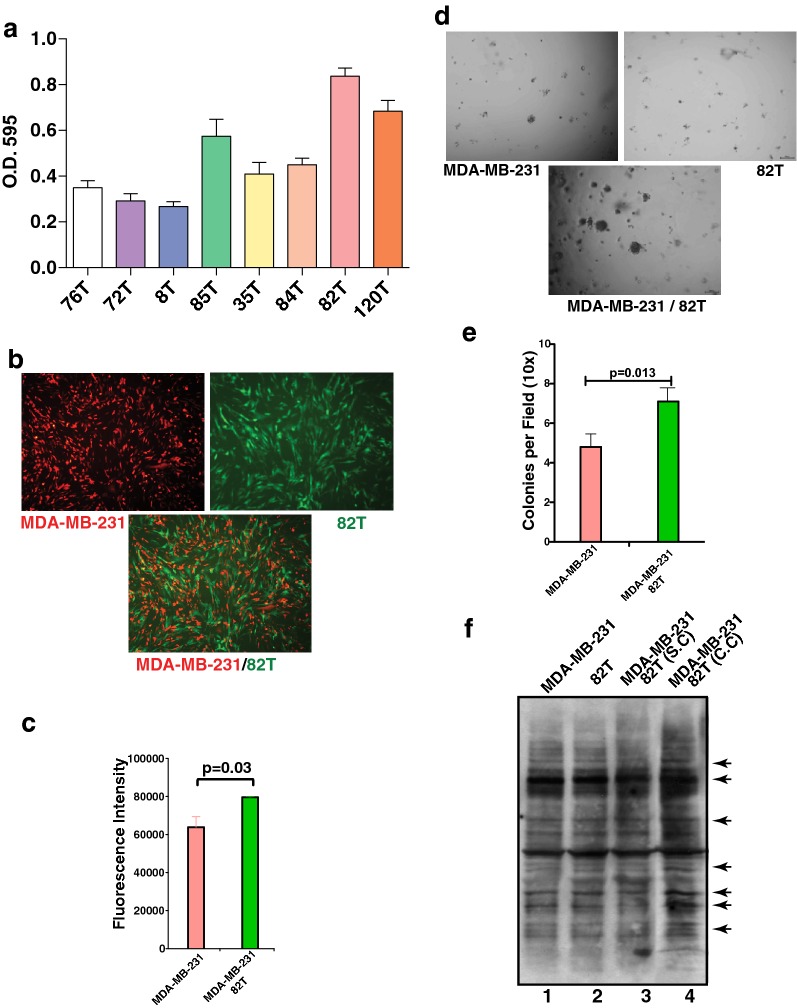
Co-culture of breast cancer cell line with CAF. **a** Growth patterns of 8 primary CAF cells. Cell proliferation assays for eight CAF cells were recorded and plotted. Y axis: average number of O.D. 595 nm of each CAF cells after crystal violet staining. Standard error of mean (SEM) bars are indicated. **b** Fluorescent microscopic image of co-cultured MDA-MB-231 (Red) with CAF 82T (Green). Bottom panel: merged image of red and green fluorescence. **c** Red fluorescence of MDA-MB-231 cells cultured with or without 82T was measured using a POLARstar Omega microplate plate reader. Student t-test was performed for assessing statistical significance. **d** Phase contrast images of soft agar colony formation assays for individually cultured and co-cultured MDA-MB-231 and 82T cells. **e** The number of colonies in each microscopic field (×10) was counted for MDA-MD-231 cells and MDA-MB-231 co-cultured with 82T cells. Y axis: average number of colonies per 10 fields. SEM bars were plotted and Student t test was used for statistical analysis. **f** Western blot analysis using 4G10 anti-phosphotyrosine antibody to survey the phosphotyrosine levels of individually cultured MDA-MB-231 (lane 1), 82T cells (lane 2) and co-cultured MDA-MB-231 and 82T cells (lane 4). Lane 3: individually cultured MDA-MB-231 and 82T cells were lysed and then mixed for phosphotyrosine western blot analysis. Arrows indicate the alteration of tyrosine phosphorylation induced by co-culture that are visible in the western blot

In order to investigate the crosstalk between epithelial tumor cells and CAFs, we used eight primary cultured CAF cell strains with early passage numbers (5–9 passages). To identify a CAF that can be efficiently SILAC labeled and can be propagated into large-scale cell culture for phosphoproteomic analysis, the growth rate of all eight CAFs was evaluated based on the cell proliferation. CAFs were cultured in 24-well dishes in DMEM/F12 medium supplemented with 10% FBS for 7 days. Crystal violet staining was performed to measure the proliferation rate of each CAF. As shown in Fig. 1a, of the eight CAFs tested, 82T and 120T CAF cells grew at a relatively faster rate and 82T had a lower passage number (p6) compared to 120T (p8). Thus, we selected 82T for this study.

### Co-culture of breast cancer cell line with CAFs

A highly aggressive triple negative breast cancer cell line, MDA-MB-231, was selected as the tumor epithelial counterpart of the CAFs. In order to differentially visualize the tumor epithelial cells and CAFs in the co-culture system, we labeled 82T cells with EGFP and MDA-MB-231 with dtTomato red fluorescence protein using retroviral or lentiviral infection. Equal number of fluorescently labeled cells were mixed and co-cultured for 5 days (Fig. 1b). The green and red fluorescence intensities were measured for 82T and MDA-MB-231 cells, respectively. The green and red fluorescence intensities of co-cultured MDA-MB-231 and 82T cells were compared to individually cultured MDA-MB-231 and 82T cells. Interestingly, we found co-culture with 82T increased the growth of MDA-MB-231 by about 25% (*p* = 0.03) compared to individually co-cultured MDA-MB-231 (Fig. 1c). However, no significant growth difference was observed between individually cultured and co-cultured 82T cells. We also evaluated the anchorage independent growth of MDA-MB-231 cells with or without 82T cells co-culture using soft agar colony formation assays. We observed that MDA-MB-231 cells co-cultured with 82T not only had significantly more colonies (Fig. 1d, e) but the colonies were also distinctly bigger compared to individually cultured MDA-MB-231 cells. These results are in line with previous reports that CAFs enhance the aggressiveness of epithelial tumor cells [23, 24].

**Fig. 2:**
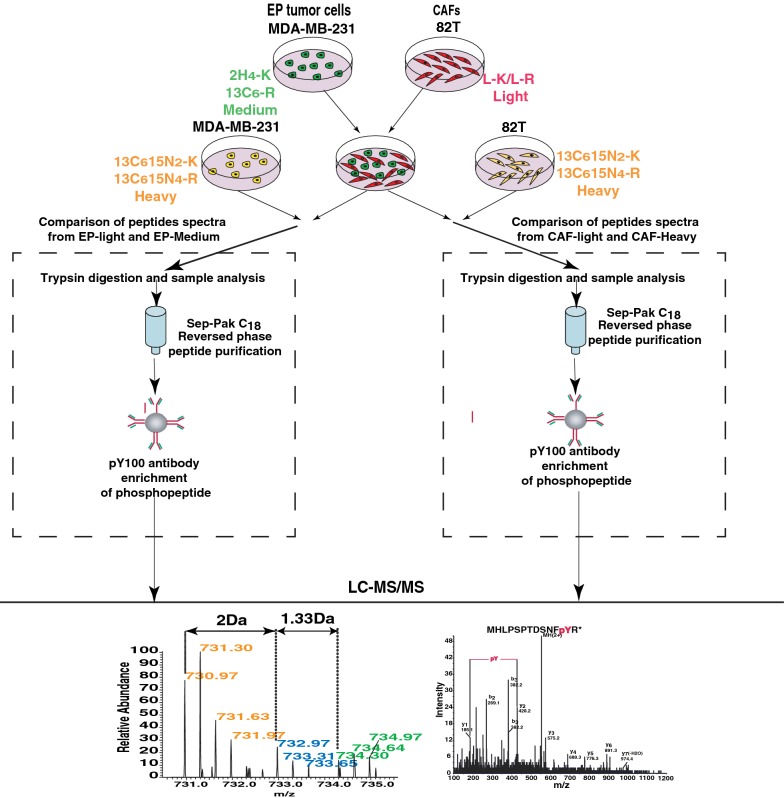
Strategy for quantitative proteomic analysis for co-cultured MDA-MB-231 and 82T cells. MDA-MB-231 cells were labeled with medium (^2^H_4_-Lys and ^13^C_6_-Arg) or heavy (^13^C_6_^15^N_2_-Lys and ^13^C_6_^15^N_4_-Arg) amino acids and 82T cells were labeled with light or heavy (^13^C_6_^15^N_2_-Lys and ^13^C_6_^15^N_4_-Arg) amino acids. Heavy-labeled MDA-MB-231 and 82T cells were individually cultured and medium-labeled MDA-MB-231 and light 82T cells were co-cultured. Individually cultured and co-cultured cells were lysed and mixed correspondingly and followed by trypsin digestion, desalting and phosphotyrosine peptide enrichment. Enriched peptides were eluted and analyzed by liquid chromatography tandem-mass spectrometry

It has been well accepted that paracrine signaling occurs between tumor epithelium and stromal fibroblasts. Growth factors secreted by tumor cells and/or CAFs can activate their receptor tyrosine kinases on recipient cells and initiate downstream signaling cascades. In order to investigate the tyrosine phosphorylation levels in cells with or without co-culture, we performed western blot analysis using an anti-phosphotyrosine antibody, which revealed that the global tyrosine phosphorylation patterns between co-cultured cells (Lane 4) and individually cultured cells (Lane 1 and 2) or individually cultured cells mixed after lysis (Lane 3) were largely similar (Fig. 1f). However, we observed several proteins whose tyrosine phosphorylation level was substantially increased due to co-culture (Fig. 1f indicated by arrows). Nevertheless, with classical western blot analysis, we could not determine the identity of these proteins and whether they were from the tumor epithelium or CAFs. In order to comprehensively decipher these signaling events, we decided to employ a SILAC labeled co-culture strategy for quantitative phosphoproteomic analysis (Fig. 2).

### SILAC labeling-based co-culture system

To identify protein phosphorylation-based signal transductions in tumor epithelium and CAFs when they are in contact, we incorporated SILAC labeling technology into the co-culture system (Fig. 2). A three-state SILAC labeling strategy was used as shown in Fig. 2. After complete labeling was achieved, we seeded medium-labeled MDA-MB-231-M on each tissue culture dish 8 h before co-culture, and an equal number of heavy-labeled MDA-MB-231-H were also seeded at the same time to serve as the individually cultured control. We detached 82T cells with enzyme free cell dissociation buffer to avoid trypsin digestion that can cleave cell surface proteins. Light-labeled 82T-L cells were seeded on each plate that had been pre-seeded with an equal number of MDA-MB-231cells. In order to detect early phosphorylation signaling events in co-cultured CAFs and breast cancer cells, we optimized the co-culture time and selected a short period of 30 min for co-culture in which CAF cells could completely settle on the plates. In parallel, heavy labeled 82T-H cells were plated and allowed to settle for 30 min, and these 82T-H cells were used as individually cultured controls. After 30 min of co-culture, the cells were harvested for mass spectrometry-based quantitative phosphotyrosine proteomic analysis.

This SILAC labeling based co-culture system allowed us to distinguish the origin of proteins by their labeling status, even when they were mixed in co-culture. For instance, in the group of individually cultured 82T-H mixed with co-cultured MDA-MB-231-M and 82T-L, we could determine that all the heavy peptides were from individually cultured 82T-H cells, light peptides were from co-cultured 82T-L cells, and medium peptides were from co-cultured MDA-MB-231-M. By comparing intensities of light peptides from co-cultured 82T-L cells with heavy peptides from individually cultured 82T-H cells (Fig. 2 right arm), we were able to identify the alterations in 82T CAFs due to the crosstalk with breast tumor cells. Conversely, by comparing medium peptides from co-cultured MDA-MB-231-M cells with heavy peptides from individually cultured MDA-MB-231-H cells (Fig. 2 left arm), we could identify the alterations in MDA-MB-231 caused by the crosstalk with CAFs, 82T cells.

### Phosphotyrosine profiling of breast cancer cells and their interacting CAFs

The majority of phosphorylation events in cells are on serine and threonine residues of proteins with a very small fraction (< 1%) occurring on tyrosine residues [25]. However, tyrosine phosphorylation is critical in relaying the extracellular signals through the activation of receptor tyrosine kinases (RTKs) into the cells [26] and tyrosine kinases play a disproportionately large role in diseases, especially in cancer. In this study, our goal was to study tyrosine phosphorylation-based signaling transduction events that were induced by the communication between tumor epithelium cells and CAFs.

**Fig. 3:**
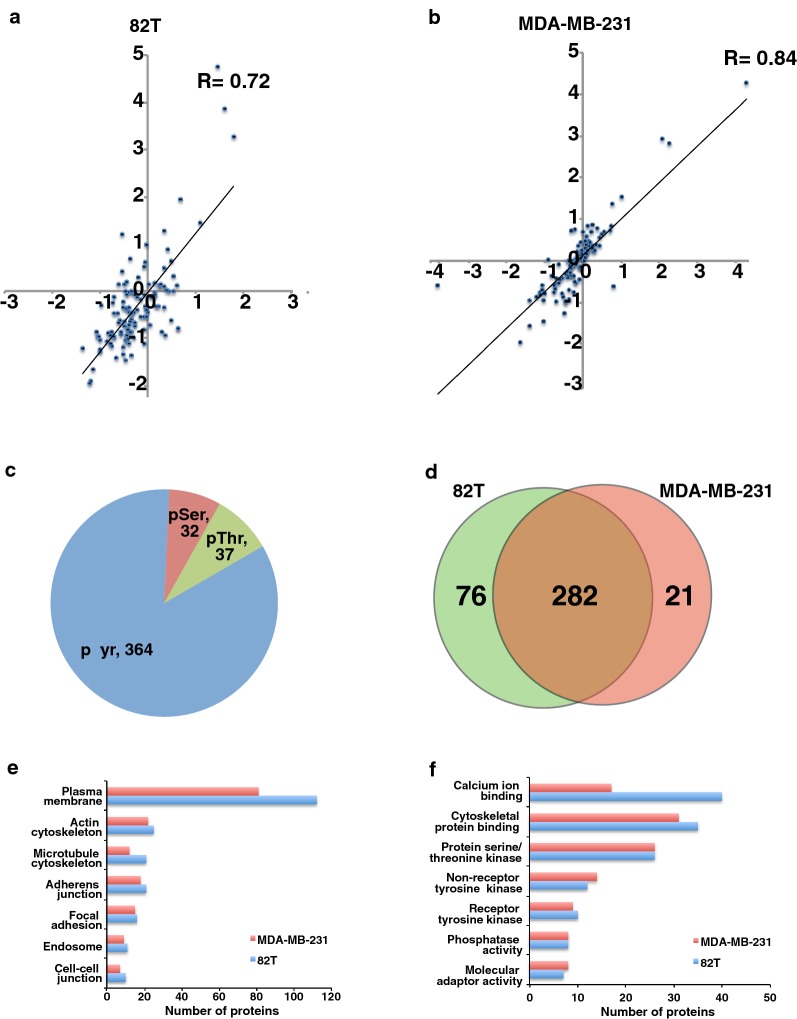
Phosphotyrosine profiling of cancer epithelial cells and interacting CAFs. **a**, **b** Density scatter plot of log_2_ transformed phosphopeptide intensity ratios (82T-co-cultured vs. 82T (A) and MDA-MB-231-co-cultured vs. MDA-MB-231) from two SILAC biological experiments. **c** Pie chart showing the composition of pTyr and pSer/Thr peptides identified in the phosphoproteomic analysis. **d** Venn diagram showing overlap of phosphopeptides identified in MDA-MB-231 and 82T cells. **e**, **f** Gene ontology analysis of phosphoproteins in cancer epithelium and CAFs. **e** Cellular component; **f** molecular functions

In order to globally examine the tyrosine phosphorylation level changes, the harvested cell lysates from co-cultured and individually cultured cells were trypsin digested, desalted and phosphotyrosine peptides were enriched using anti-phosphotyrosine antibody (pY100). The enriched peptides were analyzed using tandem mass spectrometry on LTQ Orbitrap Velos interfaced with nano liquid chromatography. Mass spectrometry analysis identified 601 unique phosphopeptides. After applying phosphoRS filtering to remove ambiguously assigned phosphopeptides, we identified 424 phosphopeptides corresponding to 291 proteins from two biological replicates. Among them, 364 peptides contain phosphorylated tyrosine, 32 peptides contain phosphorylated serine and 37 peptides contain phosphorylated threonine (Fig. 3c). The SILAC ratios (82T co-cultured cells versus 82T individually cultured cells and MDA-MB-231 co-cultured cells versus MDA-MB-231 individually cultured cells) of phosphopeptides obtained from the two independent biological replicate experiments showed a positive correlation (R = 0.72 for 82T group and R = 0.84 for the MDA-MB-231 group) (Fig. 3a, b). Among the 424 phosphopeptides, 303 phosphopeptides from 191 proteins are identified in MDA-MB-231 cells, and 358 phosphopeptides from 258 proteins are identified in 82T cells, and there are 282 phosphopeptides shared by both types of cells (Fig. 3d). We performed gene ontology analysis using the online annotation tool DAVID [27, 28], and found that the largest class of the identified phosphoproteins is associated with the plasma membrane in both tumor epithelium and CAFs cells (Fig. 3e). Forty-seven proteins identified in 82T and 48 proteins in MDA-MB-231 cells were enzymes with protein kinase activity and 10 from 82T and 9 from MDA-MB-231 cells were receptor tyrosine kinases (Fig. 3f).

### Distinct signaling pathways activated in breast cancer epithelial cells and CAFs

**Fig. 4:**
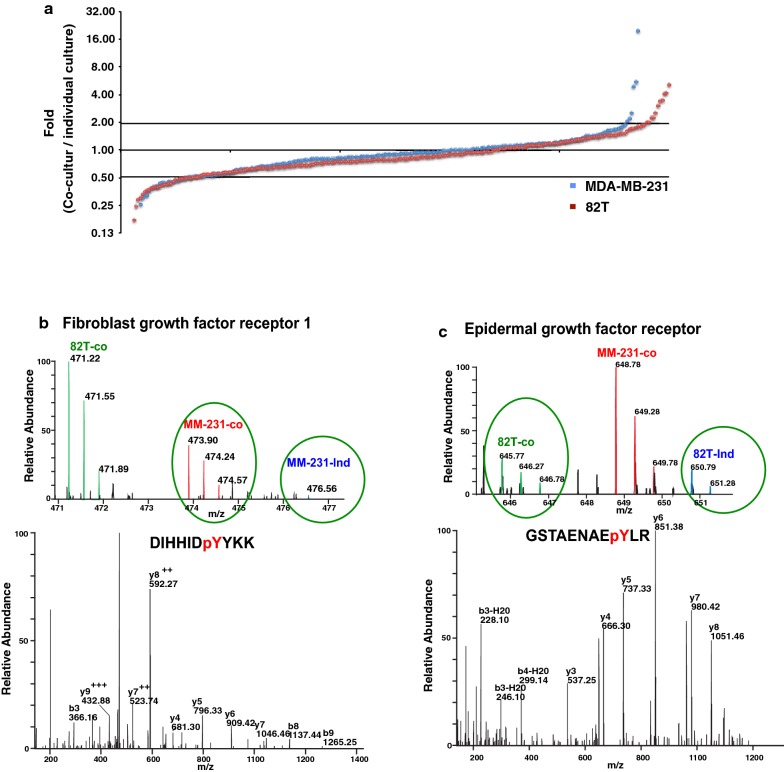
Reciprocal activation of receptor tyrosine kinases induced by the crosstalk. **a** Distribution of phosphorylation ratio of pY peptides. Blue dots: log 2 transformed ratio of MDA-MB-231-co-cultured versus MDA-MB-231; Red dots: log 2 transformed ratio of 82T-co-cultured versus 82T. **b**, **c** Representative spectrum of FGFR1 (**b**) and EGFR (**c**) identified in cancer epithelium and CAFs. Top panels: MS spectra and bottom panels: MS/MS spectra for phosphotyrosine-containing peptides identified for FGFR1 and EGFR

In order to interrogate the regulation of protein phosphorylation during the co-culture, we plotted the distribution of phosphorylation ratios (co-cultured MDA-MB-231-M vs. individually cultured MDA-MB-231-H, and co-cultured 82T-L vs. individually cultured 82T-H) in Fig. 4a. There are 51 phosphopeptides from MDA-MB-231 cells and 71 phosphopeptides from 82T cells that were regulated by more than twofold, which could be ascribed to the crosstalk that occurred between the two cell types. Of note, the majority (around 75%) of phosphopeptides identified showed no change in levels during the co-culture. This validated our SILAC labeled co-culture strategy that the peptide ratios can be preserved very well through the experimental procedures, and more importantly, the identified altered protein phosphorylation events are very likely to be real early events for the crosstalk between tumor cells and CAFs. More intriguingly, even though about 70% of identified phosphopeptides were shared between both cell types, only 7 out of 122 (~ 6%) regulated phosphotyrosine peptides were found to be regulated in both tumor epithelium and CAFs during the crosstalk. These data strongly suggest that signaling events that are activated by this crosstalk are very different in MDA-MB-231 and 82T cells (Table 1).

**Table 1:** List of upregulated pY peptides in response to the intercellular crosstalk

Phosphopeptide	Gene symbol	Protein name	82T co-culture versus individually cultured 82T	231 co-culture versus individually cultured 231
ASAyYPSSFPK	*TMEM119*	Transmembrane protein 119	Only in Co-cultured	
CCCIyEKPR	*PPP1R11*	Protein phosphatase 1, regulatory (inhibitor) subunit 11	Only in Co-cultured	0.36
DIYETDyYRK	*IIGF1R*	Insulin receptor|| insulin-like growth factor 1 receptor	Only in Co-cultured	
FGyHIIMVEGR	*PIN4*	Peptidylprolyl cis/trans isomerase, NIMA-interacting 4	Only in Co-cultured	
GASQAGMtGYGRPR	*TAGLN*	Transgelin	Only in Co-cultured	
GIVVyTGDR	*ATP1A1*	ATPase, Na +/K + transporting, alpha 1 polypeptide	Only in Co-cultured	
GKSDPyHATSGALSPAK	*GJA1*	Gap junction protein, alpha 1, 43 kDa	Only in Co-cultured	
GSTAENAEyLR	*EGFR*	Epidermal growth factor receptor	Only in Co-cultured	1.94
IEKIGEGtyGVVYK	*CDK1*	Cyclin-dependent kinase 1	Only in Co-cultured	
IGEGTYGVVyKGR	*CDK1*	Cyclin-dependent kinase 1	Only in Co-cultured	0.91
IGEGTyGVVYKGR	*CDK1*	Cyclin-dependent kinase 1	Only in Co-cultured	1.00
IGVVGGCQEyTGAPYFAAISALK	*CARKD*	Carbohydrate kinase domain containing	Only in Co-cultured	
LVQAAQMLQSDPYsVPAR	*VCL*	Vinculin	Only in Co-cultured	
NTPyKTLEPVKPPTVPNDYMTSPAR	*ABI1*	Abl-interactor 1	Only in Co-cultured	
NVPEIAVYPAFEAPPQYVLPTyEMAVK	*LAPTM4A*	Lysosomal protein transmembrane 4 alpha	Only in Co-cultured	
NVPEIAVYPAFEAPPQYVLPtYEMAVK	*LAPTM4A*	Lysosomal protein transmembrane 4 alpha	Only in Co-cultured	
QASEQNWANySAEQNR	*GJA1*	Gap junction protein, alpha 1, 43 kDa	Only in Co-cultured	
QPAPKPEPSFSEYAsVQVPR	*SIRPA*	Signal-regulatory protein alpha	Only in Co-cultured	
QPAPKPEPSFSEyASVQVPR	*SIRPA*	Signal-regulatory protein alpha	Only in Co-cultured	
QQPTQFINPETPGyVGFANLPNQVHR	*SPET2*	Septin 2	Only in Co-cultured	
QSPEDVyFSKSEQLKPLK	*EPHA2*	EPH receptor A2	Only in Co-cultured	0.95
SLyDRPASyKK	*PDGFRA*	Platelet-derived growth factor receptor, alpha polypeptide	Only in Co-cultured	
VVQEYIDAFSDyANFK	*PTPRA*	Protein tyrosine phosphatase, receptor type, A	Only in Co-cultured	1.06
YEMASNPLyR	*ITGB5*	Integrin, beta 5	Only in Co-cultured	
TYVDPHTyEDPNQAVLK	*EPHA2*	EPH receptor A2	6.60	1.29
KTPQGPPEIySDTQFPSLQSTAK	*CDV3*	CDV3 homolog (mouse)	5.16	0.51
GSHQISLDNPDyQQDFFPK	*EGFR*	Epidermal growth factor receptor	4.16	0.93
IGEGTYGVVyK	*CDK3|| CDK2|| CDK1*	Cyclin-dependent kinase1,2,,3	4.04	1.20
EATQPEPIyAESTK	*SGK223*	Homolog of rat pragma of Rnd2__	3.22	0.95
IGEGTyGVVYK	*CDK3|| CDK2|| CDK1*	Cyclin-dependent kinase1,2,,3	3.04	
IEKIGEGtYGVVYK	*CDK1*	Cyclin-dependent kinase 1	2.99	
STLQDSDEySNPAPLPLDQHSR	*LPXN*	Leupaxin	2.86	
IEKIGEGTyGVVYK	*CDK1*	Cyclin-dependent kinase 1	2.82	
IGEGTyGTVFK	*CDK5*	Cyclin-dependent kinase 5	2.77	0.79
VLEDDPEATyTTSGGKIPIR	*EPHA2*	EPH receptor A2	2.29	1.30
NSFNNPAyYVLEGVPHQLLPPEPPSPAR	*INPPL1*	Inositol polyphosphate phosphatase-like 1	2.28	0.00
TTEDEVHICHNQDGySYPSR	*LDLR*	Low density lipoprotein receptor	2.24	1.67
NTyNQTALDIVNQFTTSQASR	*CASKIN2*	CASK interacting protein 2	2.09	1.27
AGKGESAGyMEPYEAQR	*SHB*	Src homology 2 domain containing adaptor protein B		Only in Co-cultured
AHAWPSPYKDyEVKK	*GPRC5A*	G protein-coupled receptor, class C, group 5, member A		Only in Co-cultured
DKVTIADDySDPFDAKNDLK	*SHB*	Src homology 2 domain containing adaptor protein B		Only in Co-cultured
GRGEyFAIK	*PRKCD*	Protein kinase C, delta		Only in Co-cultured
GySFTTTAER	*ACTG1|| ACTB*	Actin gamma 1|| actin, beta		Only in Co-cultured
HTDDEMTGYVAtR	*MAPK14*	Mitogen-activated protein kinase 14		Only in Co-cultured
LDTASSNGYQRPGsVVAAK	*ARHGAP42*	Rho GTPase activating protein 42		Only in Co-cultured
LKQPADCLDGLyALMSR	*AXL*	AXL receptor tyrosine kinase		Only in Co-cultured
NIySDIPPQVPVRPISYTPSIPSDSR	*FAT1*	FAT atypical cadherin 1		Only in Co-cultured
NNYALNTTATYAEPYRPIQyR	*PKP4*	Plakophilin 4		Only in Co-cultured
YLNRNyWEK	*HGS*	Hepatocyte growth factor-regulated tyrosine kinase substrate		Only in Co-cultured
DIHHIDyYKK	*FGFR1*	Fibroblast growth factor receptor 1	0.98	19.26
YCRPESQEHPEADPGSAAPyLK	*STAT3*	Signal transducer and activator of transcription 3	0.98	7.03
SEQLKPLKTyVDPHTYEDPNQAVLK	*EPHA2*	EPH receptor A2		5.60
YCRPESQEHPEADPGAAPyLK	*STAT3*	Signal transducer and activator of transcription 3	0.86	4.89
NSNSYGIPEPAHAyAQPQTTTPLPAVsGSPGAAITPLPSTQNGPVFAK	*CRKL*	v-crk avian sarcoma virus CT10 oncogene homolog-like		2.49
STyTSYPK	*HGS*	Hepatocyte growth factor-regulated tyrosine kinase substrate	1.46	2.33

### Reciprocal activation of RTKs in breast cancer epithelial cells and CAFs

Our quantitative phosphoproteomic study identified 10 receptor tyrosine kinases (RTKs), which are all known to be key regulators in transmitting extracellular stimuli into cells and initiating intracellular signaling. We observed that the phosphorylation level of FGFR1-Y684 was increased 19-times in co-cultured MDA-MB-231 cells compared to individually cultured MDA-MB-231 cells. However, the phosphorylation level of FGFR1-Y684 did not change in 82T cells in co-culture compared to individually cultured cells (Fig. 4b). Y684 is located in the kinase domain of FGFR1 and is conserved among all FGF receptors. Y684 can be autophosphorylated upon the treatment of FGF and this autophosphorylation is essential for the kinase activity of FGFR1 [29]. Conversely, we also found that phosphorylation levels of EGFR Y1172 and Y1197 were dramatically elevated in 82T cells due to the crosstalk (Fig. 4c) although EGFR phosphorylation levels in MDA-MB-231 were not substantially altered. Both tyrosine sites, Y1172 and Y1197, are in the C-terminal autophosphorylation domain of EGFR. Y1172 and Y1197 are two major autophosphorylation sites of EGFR, which are critical for EGFR to induce ERK signaling through recruitment of SHC and GRB2 [30, 31]. These data point to a paracrine signal transduction between CAFs and tumor epithelium in that FGFs secreted by CAFs can activate FGFR expressed on tumor epithelial cells, and EGF secreted by tumor cells can, in turn, activate EGFR on CAFs. Additionally, we detected phosphorylated RTKs including PDGFRA and IGF1R in 82T cells co-cultured with MDA-MB-231 cells and AXL in MDA-MB-231 cells co-cultured with 82T cells. However, the phosphorylation of these RTKs was not detected in individually cultured 82T or MDA-MB-231 cells (Table 1). These are excellent examples demonstrating the existence of paracrine crosstalk between tumor epithelium and CAFs.

In addition to the RTKs activated on only one type of interacting cells, we also found an interesting case to illustrate the use of this novel system for the detection of mutual activation of RTKs induced by physical contact between tumor epithelium and CAF cells. We observed hyperphosphorylation of EPHA2 on tyrosine 588 in co-cultured MDA-MB-231 cells and tyrosine 594 and 772 in co-cultured 82T cells (Table 1). Y588 and Y594 reside in the juxtamembrane region and Y772 resides in the kinase domain of EPHA2. They are the major autophosphorylation sites of activated EPHA2 and play important roles in recruiting multiple signaling adaptor proteins including Vav2/3 GEFs, p85 and Grb7 [32, 33]. Unlike most other RTKs, both ligands and receptors for EPH family members are membrane-bound proteins (transmembrane or GPI-anchored) [34, 35]. The activation of EPHA2 in both CAF and epithelial tumor cells suggests a physical contact between CAFs and epithelial tumor, and such findings can be revealed using a SILAC-based co-culture system but not by a conventional conditioned medium system.

Besides identification of reciprocally activated RTKs, our analysis also revealed elevated phosphorylation levels of a G protein-coupled receptor, GPRC5A, on tyrosine 350 located in its C-terminal tail. Indeed, this tyrosine residue in GPRC5A has been shown to be phosphorylated by RTKs including EGFR to suppress its tumor suppressor function [36]. In our study, we found Y350 of GPRC5A was hyperphosphorylated in co-cultured MDA-MB-231 cells.

Besides increased phosphorylation level of many transmembrane proteins, we also observed that multiple cyclin dependent kinase (CDK) proteins were hyperphosphorylated in CAFs through the contact with tumor cells (Table 1). This suggests dramatic cell cycle alterations in CAFs, which is in line with published literature showing that co-culturing CAFs with MDA-MB-231 cells can accelerate CAF cell proliferation [3, 37]. More intriguingly, we discovered, for the first time, that interaction between CAFs and MDA-MB-231 cells could greatly induce hyperphosphorylation of STAT3 and PTPN11/SHP2 (Table 1). Both of these proteins play pivotal roles in mediating cell survival and transformation [38, 39] as well in developing resistance to EGFR inhibitors [40, 41]. Additionally, several non-receptor kinases and phosphatases such as PRKCD, PPP1R11, CRKL and PTPRA4 were identified for the first time as implicated in the crosstalk between CAFs and tumor epithelial cells (Table 1).

## Discussion

Communication and interaction between cells is essential for tissue homeostasis and frequently dysregulated in cancers. Although there is great progress in technology development to unravel the intracellular signaling networks using monolayer cell culture models, our ability to biochemically decipher the crosstalk between cells in direct contact remains limited. This is mainly because it is technically difficult to distinguish cell-specific proteins when different types of cells are mixed in co-culture and processed together. To achieve this goal, we employed a quantitative proteomic strategy by integrating SILAC labeling method with the classical co-culture model system coupled with mass spectrometry-based phosphoproteomic profiling to systematically and quantitatively explore signaling mechanisms underlying the crosstalk between tumor epithelium and CAFs. With this strategy, we were able to distinguish sources of individual proteins from tumor cells or CAFs even after they were mixed for co-culture and lysed together.

Using this approach, we showed that the crosstalk between CAFs and tumor cells can induce tyrosine phosphorylation of different signaling proteins in CAFs or in tumor cells. We demonstrated that the crosstalk can reciprocally activate multiple RTKs in CAFs and tumor cells. Particularly, we were able to detect unique activation of EGFR and IGF1R in CAF cells due to the communication with tumor cells. We also detected FGFR1 and AXL activation in tumor cells resulting from the communication with CAFs [42]. A recent study showed that CAF-derived GAS6, an AXL ligand, can activate AXL and promote tumor cells migration. More importantly, we also discovered that the crosstalk could bi-directionally activate EPHA2 in both tumor cell and CAFs, suggesting the direct physical interaction between CAFs and MDA-MB-231 cells. In addition to these RTKs, a number of downstream non-receptor kinases and adaptor proteins were also shown to be regulated by the crosstalk. These results indicate that not only cellular membrane receptors such as RTKs but also downstream signaling molecules are activated by the crosstalk.

## Conclusions

In this study, we used a rapidly growing CAF co-cultured with an aggressive triple negative breast cancer cell line as a model system to investigate the crosstalk signaling between these two types of cells. However, this is the first study using SILAC coupled with mass spectrometry-based proteomics to decode the bi-directional phosphorylation signaling events between breast cancer cells and their interacting primary CAFs. We believe the knowledge obtained from our exploratory study will not only facilitate the understanding of how CAFs co-evolve with tumor cells and in-turn enhance tumor aggressiveness, but also will help to develop novel therapeutic approaches to effectively disrupt the crosstalk between CAFs and tumor cells and suppress tumor growth. Finally, this model system could be used as a prototype for the analysis of intercellular crosstalk in many different tumor microenvironments, which will greatly benefit the understanding of tumor biology and ultimately accelerate the eradication of cancers.
