# Correction to: Quantitative phosphoproteomic analysis reveals reciprocal activation of receptor tyrosine kinases between cancer epithelial cells and stromal fibroblasts

**DOI:** 10.1186/s12014-018-9210-4

**Published:** 2018-11-17

**Authors:** Xinyan Wu, Muhammad Saddiq Zahari, Santosh Renuse, Nandini A. Sahasrabuddhe, Raghothama Chaerkady, Mi-Sik Kim, Mary Jo Fackler, Martha Stampfer, Edward Gabrielson, Saraswati Sukumar, Akhilesh Pandey

**Affiliations:** 1 Department of Biological Chemistry, Johns Hopkins University, Baltimore, MD USA; 2 McKusick-Nathans Institute of Genetic Medicine, Johns Hopkins University, Baltimore, MD USA; 3 Institute of Bioinformatics, International Technology Park, Bangalore, 560066 India; 4 Manipal Academy of Higher Education, Manipal, Karnataka 576104 India; 5 Department of Oncology, Johns Hopkins University School of Medicine, Baltimore, MD 21205 USA; 6 Department of Pathology, Johns Hopkins University School of Medicine, Baltimore, MD 21205 USA; 7 Division of Biological Systems and Engineering, Lawrence Berkeley National Laboratory, Berkeley, CA USA; 8 Johns Hopkins University, 733 N. Broadway, Baltimore, MD 21205 USA

## Correction to: Clin Proteom (2018) 15:21 10.1186/s12014-018-9197-x

Unfortunately, after publication of this article [1], errors were noticed in Figs. 3 and 4. The “T” in the word “pTyr” was missing in Fig. 3. The word “change” was missing after the word “Fold” in the label of y axis in Fig. 4a. The “e” in the word “Co-culture” was missing in Fig. 4a. The correct figures are presented in this correction. The original article has also been updated.

**Fig. 3:**
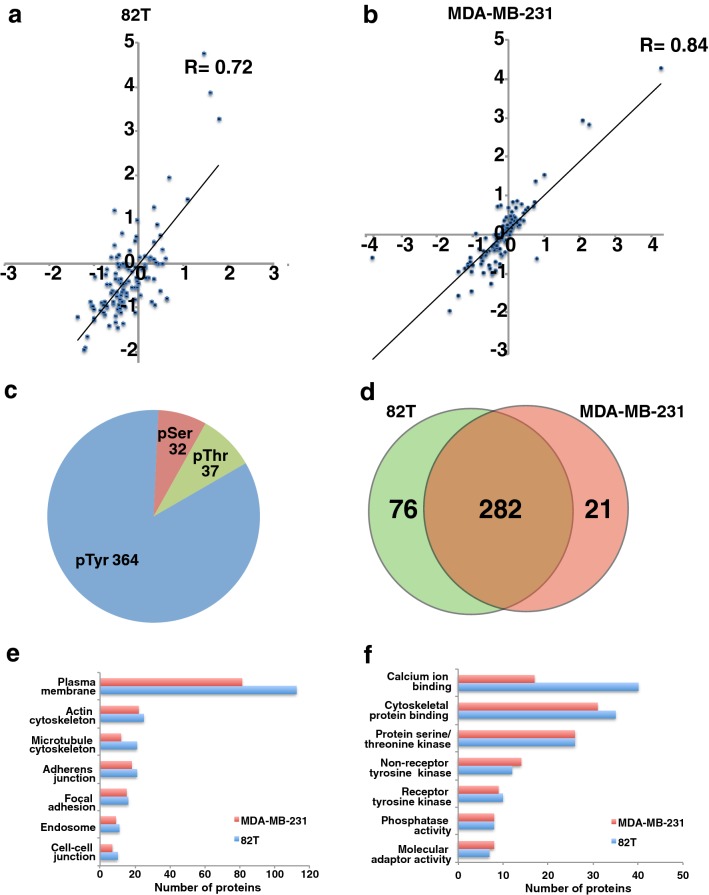
Phosphotyrosine profiling of cancer epithelial cells and interacting CAFs. **a**, **b** Density scatter plot of log_2_-transformed phosphopeptide intensity ratios (82T-co-cultured vs. 82T (A) and MDA-MB-231-co-cultured vs. MDA-MB-231) from two SILAC biological experiments. **c** Pie chart showing the composition of pTyr and pSer/Thr peptides identified in the phosphoproteomic analysis. **d** Venn diagram showing overlap of phosphopeptides identified in MDA-MB-231 and 82T cells. **e**, **f** Gene ontology analysis of phosphoproteins in cancer epithelium and CAFs. **e** Cellular component; **f** molecular functions

**Fig. 4:**
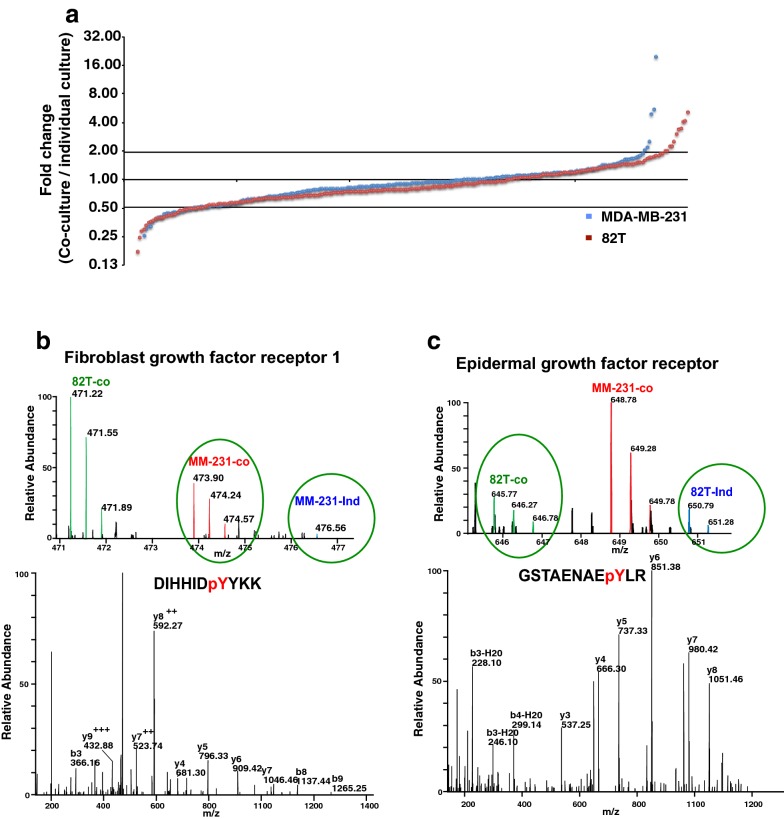
Reciprocal activation of receptor tyrosine kinases induced by the crosstalk. **a** Distribution of phosphorylation ratio of pY peptides. Blue dots: log_2_-transformed ratio of MDA-MB-231-co-cultured versus MDA-MB-231; red dots: log_2_-transformed ratio of 82T-co-cultured versus 82T. **b**, **c** Representative spectrum of FGFR1 (**b**) and EGFR (**c**) identified in cancer epithelium and CAFs. Top panels: MS spectra and bottom panels: MS/MS spectra for phosphotyrosine-containing peptides identified for FGFR1 and EGFR
